# Mesenchymal Stem Cells Improve Healing of Diabetic Foot Ulcer

**DOI:** 10.1155/2017/9328347

**Published:** 2017-03-12

**Authors:** Yue Cao, Xiaokun Gang, Chenglin Sun, Guixia Wang

**Affiliations:** Department of Endocrinology and Metabolism, The First Hospital of Jilin University, Changchun 130021, China

## Abstract

Mesenchymal stem cells (MSCs), an ideal cell source for regenerative therapy with no ethical issues, play an important role in diabetic foot ulcer (DFU). Growing evidence has demonstrated that MSCs transplantation can accelerate wound closure, ameliorate clinical parameters, and avoid amputation. In this review, we clarify the mechanism of preclinical studies, as well as safety and efficacy of clinical trials in the treatment of DFU. Bone marrow-derived mesenchymal stem cells (BM-MSCs), compared with MSCs derived from other tissues, may be a suitable cell type that can provide easy, effective, and cost-efficient transplantation to treat DFU and protect patients from amputation.

## 1. Introduction

In recent years, with the rapid economic growth and the change of diet structure, the incidence of diabetes mellitus (DM) increased gradually [1, 2]. According to epidemiological surveys, diabetes had spread to 422 million people worldwide by 2014 [3]. And the number of patients with DM may be more than 360 million in 2030 [4]. In addition, huge economic burden from treatment and care of DM is laid on the patients and society [5]. In the US, the cost on diagnosis of DM in 2012 was $245 billion, with a 41% increase compared with the expenditure in 2007 [6].

There is an alarming increase in the macro- and microvascular complications secondary to DM, in which DFU is one of the most common complications. Statistical data has demonstrated that more than a quarter of patients suffered from DFU [7]. According to the International Working Group on Diabetic Foot, risk of DFU increases with increasing age, long history of DM, and high HbA1c [8]. DFU is a complex and severe clinical problem that can lead to subsequent limb amputation. The amputation rate of DM was 19.03% in China in 2015 [9]. At present, patients with DFU are still bearing a high risk of amputation and high costs of treatment and care [10]. In summary, DFU is one of the leading factors that threaten human health and aggravate economic burden [11].

Diverse sources and the potential of self-renewing and multidifferentiation are main characteristics of stem cells, which make stem cell therapy a new alternative to repair and regenerate tissues. Nowadays, a growing number of diseases can be improved via wide applications of stem cell transplantation, such as congenital cataract [12], diabetic retinopathy and keratopathy [13], myocardial infarction [14], ocular surface burns [15, 16], serious skin burns [17, 18], Parkinson's disease [19], Huntington's disease [20], and especially DFU [21]. Accumulating evidence has pointed out that mesenchymal stem cells (MSCs) may enhance wound healing [22–24] and be served as a cell source for many tissue engineering applications including bone regeneration [25], cartilage regeneration [26–28], myocardial regeneration [29], neurogenesis [30, 31], inflammatory bowel diseases [32], and DFU [33, 34]. MSCs exist in many tissues, for example, bone marrow [35, 36], umbilical cord [37, 38], placenta [39, 40], adipose tissue [36, 41–43], gingiva [44, 45], oral mucosa [46], amniotic fluid [47], and brain [48]. However, the appropriate cell type and selection between autologous or allogeneic MSCs are yet to be discussed. Therefore, the present article reviews the roles of autologous or allogeneic MSCs derived from different tissues in wound healing of DFU.

## 2. BM-MSCs and DFU

### 2.1. Intrinsic Property

Bone marrow is one of the most common tissues from which MSCs can be acquired. BM-MSCs have no immunologic restriction and do not stimulate alloreactivity because they have capability of escaping lysis by cytotoxic T-cell and natural killer (NK) cells, reducing the formation of cytotoxic lymphocytes [49], and suppressing T-cell-derived interferon-gamma (IFN-*γ*), as well as proliferation of T-cell and NK cells induced by cellular or humoral stimuli in vitro [50, 51]. Thus, BM-MSCs transplantation is a safe way for DFU, and intramuscular transplantation has been proved to have the best efficacy [22]. However, the number and differentiated potential of BM-MSCs decline with aging [52].

### 2.2. Mechanism

#### 2.2.1. Paracrine

BM-MSCs can enhance the migration, angiogenesis, and reepithelialization via paracrine to accelerate wound repair.

Allogeneic BM-MSCs can migrate and home to the wound area [22] through expressing C-C chemokine receptor type 7 (CCR7) [53] and adhere to endothelial cells (ECs) via intercellular adhesion molecule 1- (ICAM1-), vascular adhesion molecule 1- (VCAM1-), and Akt-dependent mechanism [54].

Wan and colleagues found that allogeneic BM-MSCs could promote angiogenesis and thicken granulation tissue by increasing the expression of vascular endothelial growth factor (VEGF) in diabetic rats [22]. O'Loughlin and colleagues indicated that allogeneic BM-MSCs seeded in a collagen scaffold could improve wound healing by augmenting angiogenesis in diabetic rabbit ear ulcer model [55]. In the study of diabetic mice, neurotrophin-3- (NT-3-) stimulated human BM-MSCs in the biological tissue material expressed VEGF, nerve growth factor (NGF), brain-derived neurotrophic factor (BDNF), and other vascular endothelial factors to upregulate angiogenesis and thicken granulation tissue [56]. Coincidentally, the conditioned medium of heme oxygenase-1- (HO-1-) overexpressing human BM-MSCs promoted the proliferation and migration of human umbilical vein endothelial cells (HUVECs) in vitro. Therefore, the complex of HO-1 -overexpressing human BM-MSCs and collagen biomaterials also could promote angiogenesis and stimulate wound cicatrization in the mice of diabetic ischemic ulcer [57].

In addition, allogeneic BM-MSCs prestimulated with EGF stimulate the neovascularization through the modulation of vascular endothelial growth factor A (VEGF-A), endothelial nitric oxide synthase (eNOS), hypoxia inducible factor (HIF), and VEGF/VEGF receptor pathways in diabetic mice, thereby enhancing the recovery of blood flow [54].

**Figure 1:**
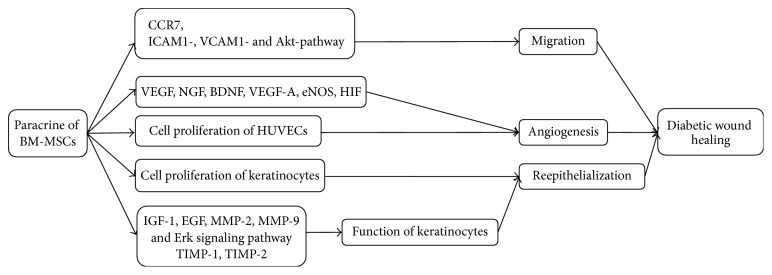
Mechanism of BM-MSCs for treatment of DFU. BM-MSCs can migrate and adhere via CCR7, ICAM1-, VCAM1-, and Akt- dependent mechanism and enhance angiogenesis through increasing VEGF, NGF, BDNF, VEGF-A, eNOS, and HIF. Cell proliferation of HUVECs and keratinocytes plays significant role in angiogenesis and reepithelialization, respectively. Keratinocyte function is improved by regulating IGF-1, EGF, MMP-2, MMP-9, TIMP-1, TIMP-2, and Erk signaling pathway. CCR7: C-C chemokine receptor type 7, ICAM1: intercellular adhesion molecule 1, VCAM1: vascular adhesion molecule 1, VEGF: vascular endothelial growth factor, NGF: nerve growth factor, BDNF: brain-derived neurotrophic factor, VEGF-A: vascular endothelial growth factor A, eNOS: endothelial nitric oxide synthase, HIF: hypoxia inducible factor, IGF-1: insulin-like growth factor 1, EGF: epidermal growth factor, MMP-2: matrix metalloproteinase-2, MMP-9: matrix metalloproteinase-9, TIMP-1: tissue inhibitor of metalloproteinase-1, and TIMP-2: tissue inhibitor of metalloproteinase-2.

Reepithelialization in the wound is a consequence of cell proliferation and modification of keratinocyte functions. Allogeneic BM-MSCs shortened the duration of wound healing in diabetic foot ulcerations on the plantar skin of rats via the improvement of keratinocytes which had been mentioned in vitro [33]. It has been found that BM-MSCs isolated from rats not only promote human keratinocytes (HKCs) to produce cytokine including matrix metalloproteinase-2 (MMP-2), epidermal growth factor (EGF), and insulin-like growth factor 1 (IGF-1) [33], but also enhance the migration and proliferation of rat keratinocytes by upregulating MMP-2 and matrix metalloproteinase-9 (MMP-9) and downregulating tissue inhibitor of metalloproteinase-1 (TIMP-1) and tissue inhibitor of metalloproteinase-2 (TIMP-2), as well as triggering Erk signaling pathway [58] (Figure 1).

#### 2.2.2. Mobilization of Autologous Stem Cells

Iwamoto and colleagues demonstrated that autologous stem cells mobilized from bone marrow by systemic injections of granulocyte colony-stimulating factor (GCSF) improved wound bed preparation and accelerated healing in mice [59]; albeit the presence of BM-MSCs in mobilized stem cells was not identified in this study, they were shown to be mobilized by GCSF in previous study [60]. In Tatsumi et al. study, GCSF might promote bone marrow-derived stem cells to mobilize and migrate to the wound site and improve granulation tissue to enhance epithelialization, rather than exerting a direct effect on epithelialization of wounds in both mice and human without diabetes. However, GCSF was unable to enhance epidermal migration from the wound margins in db/db diabetic mice with tail wounds.

The reasons are likely that diabetic microenvironment including hyperglycemia and persistence of inflammation may have an influence on population and function of endogenous stem cell. Compared to wild-type mice, db/db diabetic mice possessed fewer MSCs, of which viability, homing capacity, and therapeutic capacity were impaired [61]. Clinical trials showed that the number of MSCs was decreased and the phenotype of MSCs was altered in patients with diabetic foot syndrome [62]. Based on minimal criteria to define human MSCs [63], MSCs are defined as positive for CD105, CD73, and CD90 and negative for CD45, CD34, CD14 or CD11b, CD79*α* or CD19, and HLA-DR surface markers. However, CD45^−^, CD29^+^, and CD90^+^ MSCs were increased in subjects with diabetic foot syndrome [62]. Moreover, migration process was compromised as a result of less expression of adhesion molecules, such as ICAM1 and VCAM1 [54].

### 2.3. The Safety and Efficacy of Clinical Trials

In clinical trials, autologous transplantation of BM-MSCs can significantly ameliorate clinical parameters including decrease in wound size and increase in pain-free walking distance and maintain normal liver and renal function following intervention [64]. Leg perfusion is also sufficiently improved to minimize major amputations [65, 66]. It has been discovered that autologous biograft in combination with BM-MSCs decreases wound size and increases dermal vascularity and thickness in patients with DFU [67].

At 6 weeks after intramuscular injection of autologous BM-MSCs, the ulcer healing rate of T2DM patients with bilateral critical limb ischemia (CLI) and foot ulcer increased significantly. After 24 weeks of follow-up, painless walking time, limb perfusion, ankle-brachial index (ABI), transcutaneous oxygen pressure (TcO_2_), and magnetic resonance angiography (MRA) analysis were also improved significantly [34] (Table 1).

## 3. Umbilical Cord Blood-Derived Mesenchymal Stem Cells (UCB-MSCs) and DFU

### 3.1. Intrinsic Property

UCB-MSCs have a similar morphology, cell surface antigens, and the potential of differentiation into BM-MSCs and umbilical cord-derived mesenchymal stem cells (UC-MSCs) [42, 52, 68]. Additionally, UCB-MSCs have several advantages, such as short doubling time [69], long viable time, and anti-inflammatory activity [42]. Thus, UCB-MSCs are considered as convenience and abundance seed cells for regenerative medicine.

### 3.2. Mechanism

#### 3.2.1. Paracrine

Animal studies have indicated the ability of human UCB-MSCs to prevent or cure DFU via angiogenesis and the expression of nerve growth factor (NGF) in femoral nerve innervated gastrocnemius of diabetic foot ulceration rats [70]. In vitro, human UCB-MSCs might have capacity for diabetic wound healing by producing VEGF and basic fibroblast growth factor (bFGF) [71].

### 3.3. The Safety and Efficacy of Clinical Trials

It has been reported that transplantation of allogeneic UCB-MSCs injected into the quadriceps thigh muscles of individuals with DFU improves clinical profiles. All patients following allogeneic UCB-MSCs transplantation have decreased blood glucose, insulin dosage, levels of C-reactive protein (CRP), and tumor necrosis factor *α* (TNF-*α*), as well as increased VEGF and the ratios of CD4^+^CD25 (hi) FoxP3^+^Treg/Th17 and CD4^+^CD25 (hi) FoxP3^+^Treg/Th1 cells. Moreover, the ratio of Treg/Th17 also had a correlation with the levels of VEGF and interleukin-6 (IL-6) detected in the plasma of patients [72].

However, a phase I study on patients with CLI indicated that intramuscular injection of allogeneic UCB-MSCs improved symptoms or clinical parameters with some side effects. Adverse events including whole body urticaria, diarrhea, oral ulceration, and elevation of serum creatinine level were observed in three patients; however, all conditions were resolved in short order [73].

Up to now, the application of UCB-MSCs for DFU is little. We consider that the extraction of UCB-MSCs involved in privacy and ethic may be a concern; meanwhile the cost in preservation of umbilical cord blood is very high (Table 1).

## 4. MSCs Derived from Other Tissues

Up to date, preclinical studies on adipose-derived mesenchymal stem cells (AMSCs), umbilical cord-derived mesenchymal stem cells (UC-MSCs), placenta-derived mesenchymal stem cells (PMSCs), and human amniotic fluid-derived stem cells (AF-MSCs) for diabetic wound healing have been reported, but no clinical trials have been reported. However, human gingiva-derived mesenchymal stem cells (GMSCs) are only investigated in excisional wound model, and the data are quite limited.

### 4.1. AMSCs and Diabetic Wound Healing

#### 4.1.1. Intrinsic Property

Adipose tissue derived from the mesenchyme is widely distributed and easily isolated. AMSCs have high colony frequency and represent an attractive alternative source of pluripotent cells, whose characteristics are similar to BM-MSCs [42, 74].

#### 4.1.2. Mechanism

In diabetic rats with dorsal full-thickness skin wound, allogeneic AMSCs injected subcutaneously in the wound margin stimulated neoangiogenesis and increased tissue regeneration through paracrine and autocrine mechanisms [75]. The data showed that allogeneic AMSCs migrated to the wound margin and increased angiogenesis via the activation of endothelial activity and neoangiogenic capacities by increasing VEGF and von Willebrand factor (vWF). Simultaneously, as a proliferating cell nuclear antigen, Ki-67, was up-regulated to promote cellular proliferation. The proinflammatory reaction was reduced through the expression of EGF, VEGF, and prolyl 4-hydroxylase (rPH). Consistent with this notion, allogeneic AMSCs were harvested from the inguinal fat of normal rats secreted large amounts of several angiogenic growth factors including VEGF, hepatocyte growth factor (HGF), transforming growth factor beta 1 (TGF-*β*1), IGF-1, EGF, and keratinocyte growth factor (KGF) in vitro. In vivo, the transplantation of AMSCs sheets was created using cell-sheet technology accelerated wound healing and vascularization in full-thickness skin defects in Zucker diabetic fatty rats [76].

Additionally, direct injection of ASCs obtained from nondiabetic patients into full-thickness wound of diabetic mice model significantly increased the rate of wound closure [77]. In another study on diabetic mice, the new findings that silk fibroin patches cellularized with human adipose-derived MSCs (Ad-MSCs-SF) and silk fibroin patches decellularized with human adipose-derived MSCs (D-Ad-MSCs-SF) patches improved tissue regeneration and reduced the wound area through releasing angiogenic factors and collagen deposition stimulating molecules [78]. A decrease in the risk of transferring genetically mutated cells and the possibility of stimulating the immune system were the advantage of D-Ad-MSCs-SF patches, and decellularized patches could be prepared and stored for an extended period.

### 4.2. UC-MSCs and Diabetic Wound Healing

#### 4.2.1. Intrinsic Properties

UC-MSCs are generally considered to be rich, safe, of short doubling time, and easy to collect [52]. Compared to BM-MSCs, it has been well documented that UC-MSCs have similar characteristics involving fibroblastic morphology, typical immunophenotypic markers, and multiple differentiation potential to BM-MSCs [79–82]. In addition, the trait of UC-MSCs has lower immunogenicity [83, 84].

#### 4.2.2. Mechanism

In the study on DFU rats with UC-MSCs delivered through the left femoral artery, researchers found that UC-MSCs could specifically localize to the targeted area by detecting the expression of human leukocyte antigen type-I (HLA-1), a marker to track UC-MSCs in vivo.

Besides, UC-MSCs significantly reduced the size of foot ulcers and promoted epithelialization of ulcerated tissue via release of cytokeratin 19 from keratinocytes and formation of extracellular matrix [21]. In other studies of DFU rats, the data showed that administration of UC-MSCs contributed to improvement of vascular density [85, 86] and repair of wound and sensory functions [87] by the expression of VEGF, keratinocyte growth factor (KGF), platelet derived growth factor (PDGF), and brain-derived growth factor (BDGF).

### 4.3. PMSCs and Diabetic Wound Healing

#### 4.3.1. Intrinsic Property

Placental tissue is readily available and can isolate a large number of MSCs for clinical application [88]. What is more, the morphology, size, surface phenotype, and immunosuppressive characteristics of PMSCs are similar to BM-MSCs, and the proliferation capability is better [39]. The best efficacy delivery is intraperitoneal injection [89].

#### 4.3.2. Mechanism

In the research of diabetic Goto-Kakizaki (GK) rats, the experimental group showed that implanted PMSCs gathered to the wound tissue and differentiated into endothelial-like cells. Additionally, it has been found that PMSCs participate in angiogenesis in wound bed through secreting some proangiogenic molecules, such as VEGF, bFGF, and IGF-1, transforming growth factor-*β* (TGF-*β*) and hepatocyte growth factor (HGF) [90].

### 4.4. AF-MSCs and Diabetic Wound Healing

Large numbers of human AF-MSCs can be easily harvested from as little as 2 mL of amniotic fluid [91]. Human AF-MSCs remain stable and show high proliferative capacity, multilineage differentiation potential, immunomodulatory activity, and lack of significant immunogenicity [92].

The transplantation of human AF-MSCs has been shown to accelerate wound healing by secreting factors [93] to stimulate proliferation and migration of dermal fibroblasts. In full-thickness excisional wound of diabetic NOD/SCID mice, human AF-MSCs significantly accelerated wound closure through increasing the angiogenic factors, IGF-1, EGF, and interleukin-8 (IL-8), as well as enhancing reepithelialization by expressing keratinocyte-specific proteins and cytokeratin in the wound area [94]. Additionally, in a model of mouse with excisional wound, human AF-MSCs significantly enhanced wound healing via the TGF-beta/SMAD2 pathway [95], while human AF-MSCs accelerated wound closure through TGF-*β*/SMAD2 and PI3K/Akt pathways under the condition of hypoxia [96].

### 4.5. GMSCs and Wound Healing

Human GMSCs are homogenous, not tumorigenic [97], and easy to be isolated [98] and display stable phenotype. The most significant advantage of human GMSCs is without any ethical problems in clinical application [99]. Moreover, human GMSCs show a greater capacity of proliferation and migration than AMSCs [100] and BM-MSCs without growth factors [99].

In a murine excisional full-thickness skin wound model, systemic infusion of human GMSCs mitigated local inflammation mediated via suppression of inflammatory cells infiltration, production of IL-6 and TNF-*α*, and increasing expression of interleukin-10 (IL-10) [101]. This mechanism also existed in the hypoxic environment [102]. In addition, human GMSCs have elicited M2 polarization of macrophages, which may contribute to rapid reepithelialization, improvement of angiogenesis, and tissue remodeling of skin wound [101].

## 5. Are Autologous or Allogeneic MSCs More Appropriate?

It has been shown that autologous BM-MSCs are a major source and have obvious efficacy in cell therapy for patients suffering from DFU. Most recently, a study on the feasibility of autologous stem cell therapy in diabetic patients showed that AMSCs isolated from distal limbs of diabetic patients with critical ischemia was not satisfactory as an autologous AMSC source because of its improper phenotype and function [103]. In line with above evidence, the initial viability of the mouse MSCs extracted from the bone marrow of diabetic mice was poor in a normal glucose environment in vitro, but the expansion of that was subsequently improved [61].

Although allogeneic MSCs have had potent immunosuppressive properties, evidence also suggests that they elicit potential as a new therapeutic strategy for the treatment of DFU in animal models. Moreover, allogeneic UCB-MSCs have been successfully used to treat patients with DFU. With increasing number of clinical trials of allogeneic MSCs for acute and chronic diseases [104–107], a comprehensive understanding of the difference in immunological profile is essential.

Hence, the potential for autologous or allogeneic MSCs to be used to improve diabetic wound healing appears particularly promising. However, so far preclinical and clinical data are quite limited and further studies need to be explored for the feasibility of autologous and allogeneic MSCs therapy of DFU.

## 6. The Further Treatment for DFU

Recent studies showed that a transgenic* L. sericata larvae* could secrete platelet derived growth factor-BB (PDGF-BB), a dimeric peptide growth factor that could bind to the platelet derived growth factor (PDGF) receptor and stimulate cell proliferation and survival, and, hence, promote wound healing. It may be a cost-effective manner for nonhealing wounds, especially for patients with DFU [108] and be employed in regenerative medicine strategies to enhance tissue repair.

## 7. Conclusion

A variety of clinical applications need large number of functionally competent MSCs with stable phenotype to achieve successful results.

From the above, the morphology, size, and surface phenotype of MSCs derived from different tissues have no significant difference. Besides BM-MSCs, others possess rich source and greater proliferation capability and can be easily isolated. In addition, UC-MSCs and human AF-MSCs have lower immunogenicity, while AMSCs and human GMSCs pose fewer ethical problems. Although BM-MSCs have some limitations, they are firstly discovered and deeply studied in many clinical trials with satisfactory clinical efficacy. This paper supports the potential of BM-MSCs for treatment of DFU, and it may be the optimal cell type for safe and feasible transplantation of DFU.

## Figures and Tables

**Table 1:** Clinical trials of BM-MSCs and UCB-MSCs.

First author	Publication year	Cellular type	Object	Delivery method	Duration of observation	Clinical parameters
Dash	2009	Autologous BM-MSCs	24 patients with nonhealing ulcers of the lower limb (diabetic foot ulcers and Buerger disease)	Autologous cultured BM-derived MSCs along with standard wound dressing	12 weeks	Decrease in wound size, increase in pain-free walking distance, maintain normal liver and renal function, improve leg perfusion sufficiently

Amann	2009	Autologous BM-MSCs	51 patients with impending major amputation due to severe critical limb ischemia	Intramuscular transplantation	6 months	Improve leg perfusion sufficiently to reduce major amputations and permit durable limb salvage, reduce analgesics consumption, increase in pain-free walking distance

Vojtassak	2006	Autologous biograft composed of autologous skin fibroblasts on biodegradable collagen membrane (Coladerm) in combination with autologous BM-MSCs	Patients with diabetic foot	Directly to the wound and injected into the edges of the wound, finally covered with prepared autologous biograft, received two additional treatments with cultured MSC on days 7 and 17	29 days	Decrease in wound size and an increase in the vascularity of the dermis and in the dermal thickness of the wound bed

Lu	2011	Autologous BM-MSCs	41 type 2 diabetic patients with bilateral critical limb ischemia and foot ulcer	Intramuscular injection	24 weeks	Increase in pain-free walking distance, improve leg perfusion, ankle-brachial index (ABI), transcutaneous oxygen pressure (TcO_2_), magnetic resonance angiography (MRA) analysis

Procházka	2010	Autologous BM-MSCs	96 patients with critical limb ischemia and foot ulcer	Inject into the ischemic limb along the posterior and anterior tibial artery	120 days	79% limb salvage in patients

Li	2013	Allogeneic UCB-MSCs	15 diabetic patients with foot disease	10 mL is injected intramuscularly into impaired lower limbs and 2 mL is delivered into the basilar portions of foot ulcers and the surrounding subcutaneous tissues	12 weeks	Weakness, numbness, pain, cold feeling, or intermittent limp, skin temperature, ABI, and transcutaneous oxygen pressure (TcO_2_) are improved

BM-MSCs: bone marrow-derived mesenchymal stem cells and UCB-MSCs: umbilical cord blood-derived mesenchymal stem cells.
